# Efficacy of S53P4 Bioactive Glass for the Secondary Obliteration of Chronically Discharging Radical Cavities

**DOI:** 10.1002/oto2.96

**Published:** 2023-11-29

**Authors:** Victor J. Kroon, Steven W. Mes, Pepijn A. Borggreven, Rick van de Langenberg, David R. Colnot, Jasper J. Quak

**Affiliations:** ^1^ Department of Otolaryngology and Head and Neck Surgery Diakonessenhuis Utrecht Utrecht The Netherlands; ^2^ Amsterdam UMC location Vrije Universiteit Amsterdam Amsterdam The Netherlands; ^3^ Department of Otolaryngology Cambridge University Hospitals NHS Foundation Trust Cambridge UK

**Keywords:** bioactive glass, mastoid, obliteration, radical cavity, S53P4

## Abstract

**Objective:**

Present the results of the secondary obliteration of chronically discharging radical cavities using S53P4 bioactive glass (BAG).

**Study Design:**

Retrospective cohort study.

**Setting:**

Single‐center study.

**Methods:**

A single‐center retrospective cohort study was conducted of all patients that underwent secondary obliteration of persistently draining radical cavities using S53P4 BAG between 2011 and 2022. Patients with middle ear cholesteatoma were excluded. The main outcome was postoperative otorrhea, as indicated by Merchant grading.

**Results:**

In total, 97 patients were included. The median postoperative follow‐up time was 3.9 years (range 0.5‐10.4). Average time between the original canal wall down surgery and the secondary obliteration was 25.3 years (SD 11.7, range 2‐66). At the most recent follow‐up visit, a Merchant grade of 0 to 1 was observed in 95% of the cases. There were no cases of sensorineural hearing loss or facial palsy, one case developed a retro auricular skin defect and 1 patient developed CSF leakage. Minor complications were seen in 10 patients (10%). Ossicular chain reconstruction with a titanium prosthesis was performed in 42 cases, resulting in a median improvement of 11.2 dB in air conduction thresholds. In 9/42 cases (21%), closure of the postoperative air‐bone gap to ≤20 dB was achieved. Twenty‐five percent of cases could be discharged from out‐patient visits.

**Conclusion:**

Revision of persistently draining radical cavities with BAG obliteration is feasible and results in a dry and safe ear in 95% of the patients, thereby enabling wearing of a conventional hearing aid. Out‐patient visits could be ceased in 25% of the cases.

In canal wall down surgeries, the posterior bony wall of the external auditory canal (EAC) is removed to increase exposure. The creation of a so‐called radical cavity comes with several possible disadvantages, such as higher rates of otorrhea and purulence, pain, adherence to water precautions, and dizziness.^1^, ^2^, ^3^ While most discharging radical cavities can be treated conservatively with regular cleaning and eardrops, older studies estimated that 10% to 30% will remain troublesome.^4^, ^5^ In recent years, secondary obliteration of these troublesome radical cavities is gaining popularity.^2^, ^6^ The aim is to eradicate chronic infection and to obtain a safe, dry, and waterproof ear by obliterating the mastoid and reconstructing the external auditory canal. This is achieved in 93% to 98% of the patients.^1^, ^3^, ^7^, ^8^ Additionally, several studies have demonstrated that the secondary obliteration can also results in significant quality of life (QoL) improvement.^9^, ^10^, ^11^

Various materials can be utilized as obliteration material, including autologous such as bone dust and bone pâté, allogenic such as bone chips from a donor and synthetic material such hydroxyapatite or ceramic granules.^12^, ^13^, ^14^, ^15^, ^16^ A more recent synthetic material is S53P4 bioactive glass (BAG) which has several unique properties: S53P4 BAG has an inhibitory effect on bacteria and it is osteoconductive and osteostimulative.^17^, ^18^, ^19^ The inhibitory effect on bacterial growth results from the increase in pH due to an efflux of ions after application of the BAG. In several in vitro studies, the rise in pH has been effective against various clinically relevant bacteria.^17^ The osteoconductive and osteostimulative effects are result of BAG forming a hydroxyapatite layer which also functions as scaffolding for osteoblasts.^19^ Other important properties of synthetic materials in general are the preservation of volume over time, the unlimited supply, and no risk of donor site morbidity. Therefore, since 2011, BAG has been the obliteration material of choice in our hospital, and we have operated over 800 patients using this technique.

Previous studies, including from our own hospital, have demonstrated the use of S53P4 BAG for indications such as cholesteatoma and chronic otitis media.^20^, ^21^, ^22^ Studies on secondary obliteration of old, purulent, cavities after a previous CWD surgery, without the presence of cholesteatoma, are lacking. In this study, we present the results of the use of S53P4 BAG for the secondary obliteration of troublesome radical cavities with as main outcome control of infection as graded by postoperative Merchant grade.^23^

## Methods

### Ethical Considerations

This retrospective cohort study was conducted at a secondary referral center in the Netherlands. The study was in accordance with the ethical standards of the hospital institutional review board (MEC‐U; Medical Research Ethics Committees United, Netherlands, registration number W21.162) and the 1964 Declaration of Helsinki. Formal consent was not required for this type of retrospective study. The S53P4 BAG, produced by BonAlive Biomaterials Ltd., has received clearance for clinical use by the CE in 2004 and is currently awaiting Food and Drug Administration (FDA) approval for mastoid surgery. BAG has received FDA approval for use in orthopedic surgery, mainly as filler material for bone defects such as nonunions.^24^

### Patient Characteristics

Eligible for inclusion were all patients with troublesome radical cavities due to a previous CWD surgery that underwent secondary obliteration using S53P4 BAG in the period of January 2011 to September 2022. All patients were initially treated conservatively, but symptoms would persist despite maximal treatment. Patients were excluded if the cause of the troublesome radical cavity was middle ear cholesteatoma. The patients reported in our previous publication were not included in this study, as they were diagnosed with cholesteatoma.^20^ Cholesteatoma was defined as a mass formed by keratinizing squamous epithelium in the tympanic area, in accordance with the EAONO/JOS Joint Consensus Statement by Yung et al.^25^ Keratin masses presenting solely in a former radical cavity and that were accessible by cleaning at the outpatient clinic were not considered cholesteatoma by this definition. Patients were also excluded if previous obliteration had been performed.

### Surgical Technique and Follow‐Up

All patients received a CT‐scan preoperatively for surgical planning The canal wall down tympanomastoidectomy with mastoid obliteration (CWD + MO) was utilized as surgical treatment of the troublesome radical cavities. Care was taken to remove all possible causes of the troublesome mastoid, such as infected mucosa or mastoid cells. The posterior wall of the EAC would be reconstructed with cartilage. The BAG granules would be moistened inside their container with 0.9% sterile saline solution. Subsequently, the material is carefully applied to both the epitympanic area and the mastoid cavity. Attention is paid that no cavities exist within the obliteration. Thereafter, the fluid is suctioned from the material and the obliteration is covered with periosteal or fascia flaps.^20^ Adequate and sturdy reconstruction of the posterior wall of the EAC is important to prevent displacement of BAG granules during the healing process. Follow‐up consisted of clinical otoscopy at 1 and 8 weeks following surgery, at 3‐ to 6‐month intervals for 1 year, and thereafter once yearly unless more frequent visits were deemed necessary.

### Outcome Measures

The following outcome parameters were included: indication for secondary obliteration, time between original CWD surgery and secondary obliteration, procedure safety, postoperative Merchant grade at 8 weeks, 6 months, 1 year, and most recent follow‐up moment, secondary cholesteatoma development, and audiometric performance. Possible indications for secondary obliteration were defined as the presenting symptoms of patients and included chronic discharge, dizziness, hearing improvement, and pain. Procedure safety was defined as the absence of perioperative and postoperative complications in the complete follow‐up and need for any revision surgery. Merchant's grading system was used to evaluate postoperative otorrhea (Table 1).^23^ Patients with no otorrhea or less than once in the previous three months were categorized as Merchant grade 0 to 1 (control of the infection) and patients with otorrhea more than once in the previous three months, granulation tissue or constant discharge were categorized as Merchant grade 2 to 3 (failure to control the infection). Development of secondary cholesteatoma was defined as development of a mass of keratinizing squamous epithelium invading the middle ear during postoperative follow‐up. Univariate analysis was performed to determine predictive factors for unsuccessful surgery with the following variables: years since original CWD surgery, indication of original CWD surgery, age, sex, preoperative Merchant grade, and postoperative complication. Unsuccessful surgery was defined as Merchant grade 2 to 3 at the most recent follow‐up visit or before additional revision surgery. Audiometric evaluation was performed preoperatively and postoperatively using pure‐tone audiometry (PTA) at 500, 1000, 2000, and 4000 Hz for both air and bone conduction (AC and BC, respectively) and the average AC and BC were calculated. The average air–bone gap (ABG) was calculated from the difference in AC and BC. Audiometric data were only analyzed if both preoperative and postoperative tone audiometry were complete. Additionally, word recognition score (WRS) was evaluated using a standardized word list of monosyllables of the consonant‐vowel‐consonant type (CVC), measured as a percentage of correctly recognized syllables after listening to a recorded word list in quiet.^26^ We evaluated the percentage of word recognition at 50 dB and the dB level necessary for 50% word recognition.

**Table 1:** Merchant Grading System for Otorrhea

Merchant grade 0	Dry ear, no otorrhea in the last 3 months and normal otoscopy.
Merchant grade 1	Subjective wet feeling in the ear or 1 otorrhea episodes in the last 3 months, lasting less than 2 weeks. Both without granulation tissue during otoscopy.
Merchant grade 2	Otorrhea lasting longer than 2 weeks or more than 1 episode of otorrhea in the last 3 months or granulation tissue during otoscopy.
Merchant grade 3	Continuous otorrhea.

### Statistical Methods and Data Analysis

Data were analyzed using SPSS statistics (version 27; IBM Corp.). Continuous data were presented as mean ± standard deviation (SD) or median with interquartile range (IQR), depending on normal or nonnormal distribution. Fisher's exact test, Chi‐squared test and Mann‐Whitney *U* test were used for univariate analysis. For audiometric evaluation, Wilcoxon signed‐rank test was used for comparison of preoperative and postoperative AC, BC, speech recognition, and ABG. Mann‐Whitney *U* test was used to compare postoperative ABG between different subsets of cases. Scattergrams were constructed by combining the word recognition at 50 dB and average AC.^27^ A Sankey flowchart of the Merchant grade was created using sankeymatic.com. *P* < .05 was considered statically significant.

## Results

In total, 97 cases were included in this study with a mean age of 51 years (range 13‐87, Table 2). Fifty‐one cases were operated on the left ear and 46 on the right. The indications for the original CWD surgery were cholesteatoma (n = 66), chronic otitis media (n = 11), or unknown (n = 20).

**Table 2:** Patient Characteristics, n (%)

Total cases included	97 (100)
Female	42 (43)
Age in years (mean ± SD)	51 ± 14
Time between original CWD and secondary obliteration (mean ± SD)	25.3 ± 11.7
Pediatric patients (<18 years)	2 (2)
Side	
Left	51 (53)
Right	46 (47)
Ossicular chain at the end of surgery	
Intact	4 (4)
Previous reconstruction left in place	7 (7)
Cartilage and/or fascia	14 (14)
Incus interposition	1 (1)
PORP	14 (14)
TORP	31 (32)
No reconstruction performed	26 (27)

Abbreviations: CWD, canal wall down; PORP, partial ossicular replacement prosthesis; SD, standard deviation; TORP, total ossicular replacement prosthesis.

The average time between original CWD surgery and secondary obliteration was 25.3 years (SD 11.7, range 2‐66). A previous revision of the radical cavity without obliteration had been performed in 17 cases. Indications for secondary obliteration were otorrhea (n = 94, 97%), dizziness (n = 21, 22%), pain (n = 7, 7%), improvement of hearing (n = 16, 16%), stenosis of the external auditory canal (n = 2, 2%) and severe complications of the existing radical cavity such as encephalitis (n = 1, 1%).

### Surgical Technique and Intraoperative Findings

A CWD + MO surgery was performed in all cases. During surgery, erosion of the bony covering of important structures was observed in 47 cases. Exposed dura was seen in 19 cases, exposed sigmoid sinus in 7 cases, a partially eroded labyrinth in 6 cases, and exposed facial nerve in 30 cases, of which 18 were exposed in the tympanic segment and 12 in the mastoid segment. The exposed structures would be covered with fascia or cartilage before obliteration of the mastoid cavity. Covering of the obliteration and reconstructed external auditory canal with an additional flap was conducted in 49 cases: 5 with temporal muscle, 14 with temporal muscle fascia, and 30 with a mid‐temporal artery/periosteal flap. The status of the ossicular chain at the end of surgery is displayed in Table 2.

### Merchant Grade

**Figure 1:**
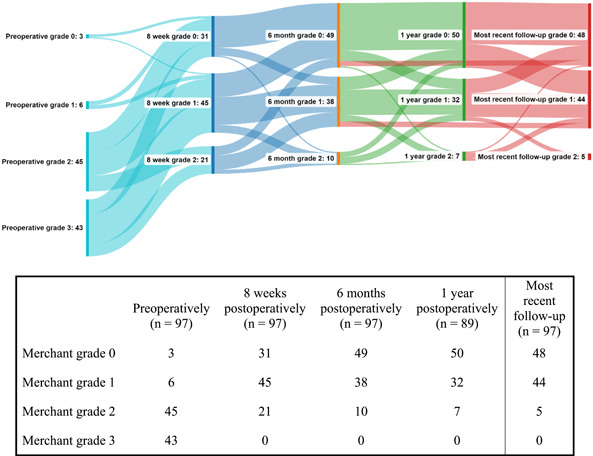
Sankey diagram and table showing the course of the Merchant grade for individual patients at different time points (preoperatively, 8 weeks, 6 months, 1 year, and most recent visit). Made using sankeymatic.com.

Preoperative and postoperative Merchant grade are presented in Figure 1, additionally showing the change in Merchant grade throughout follow‐up using a Sankey diagram. Preoperatively, 88 cases suffered from a Merchant grade of 2 to 3, while at the most recent follow‐up moment, five cases had a Merchant grade of 2 (*P* < 0.001). No cases of Merchant grade 3 were seen at the most recent follow‐up moment. This results in a dry‐ear rate of 95%. Univariate analysis was conducted of patients that developed Merchant grade 2 to 3 at the most recent follow‐up visit or before an additional surgery (Table 3). Only postoperative complications were identified as a factor significantly associated with the development of Merchant grade 2. Multivariable analysis could not be performed due to the low number of events.

**Table 3:** Univariate Analysis of Factors Prognostic for the Development of Merchant Grade 2 to 3 Postoperatively, Either at Most Recent Follow‐Up Moment or Before Additional Revision Surgery (n)

	Wet ear		
	Yes (n = 8)	No (n = 89)	*P* value
Age in years (median IQR)	54 (47‐72)	53 (36‐62)	0.412
Female	4	38	0.724
Male	4	51	0.724
Time since original CWD surgery in years (median IQR)	19 (6‐29)	24 (14‐37)	0.172
Any postoperative complication	5	7	<0.001^a^
Merchant grade preoperative 2‐3	8	80	1.000
COM as indication for original surgery	1	10	1.000
Cholesteatoma as indication for original surgery	6	60	1.000

Abbreviations: COM, Chronic Otitis Media; CWD, Canal Wall Down; IQR, Interquartile range.

^a^ Significant using Fisher's exact test.

### Postoperative complications and additional procedures

The median follow‐up time was 3.9 years (IQR 2.1‐5.9 years, range 0.5‐10.4). Following secondary obliteration out‐patient visits were ceased in 24 patients (25%). Postoperative complications were seen in 12 cases (12%), of which the majority (n = 10, 83%) was minor (Table 4). There were two patients with major complications, that is, one case developed CSF leakage and one case developed a postauricular skin defect which required intravenous antibiotics. Loss of part of the obliteration material during healing was seen in 11 cases. No patients with severe perceptive hearing loss were seen.

**Table 4:** Postoperative Complications, n (%)

Minor	
Extrusion of ossicular prosthesis	2 (17)
Stenosis of the external ear canal	1 (8)
Otorrhea requiring oral antibiotics	3 (25)
Recurrent perforation tympanic membrane	4 (33)
Major	
Skin defect due to loss of BAG during healing requiring I.V. antibiotics	1 (8)
CSF leakage	1 (8)
Total	12 (100)

Abbreviations: BAG, bioactive glass; CSF, cerebrospinal fluid; I.V., intravenous.

Some type of additional surgery was necessary in 12 cases (12%). Seven cases underwent revision surgery for the following indications: insufficient obliteration due to loss of obliteration material requiring re‐obliteration (n = 2), insufficient obliteration as insufficient material was placed during surgery requiring re‐obliteration (n = 3), CSF leakage (n = 1) or cholesteatoma (n = 1). Therefore, the revision rate of the obliteration itself was 5% (n = 5). The symptoms of patients with insufficient obliteration requiring revision varied: three cases had a small residual cavity without self‐cleaning capabilities causing recurrent infections, one case had an pre‐existent labyrinth dehiscence exposed due to insufficient obliteration which caused dizziness and one case developed a resistant skin infections, causing a skin defect and loss of the obliteration material. Removal of the obliteration material and subsequent reobliteration of the cavity was necessary in 6 of the revision surgeries. Additionally, 2 cases underwent meatoplasty and 3 patients underwent a procedure for hearing improvement. The single cholesteatoma occurred in a patient who refused middle ear surgery, therefore the epitympanum could not be obliterated and a retraction pocket developed.

### Audiological Outcomes

**Figure 2:**
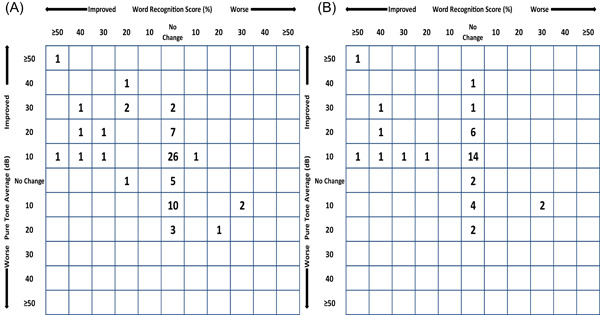
Scattergram showing the change in word recognition score and pure tone average from preoperatively to postoperatively. (A) Whole cohort (n = 69). (B) Patients that underwent PORP or TORP reconstruction (n = 40).

Complete tone audiometry was available for 78 cases (80%). The median BC did not change significantly preoperatively to postoperatively (Table 5). Both the median AC and median ABG showed minor, but significant, improvement (Table 5). When solely examining patients that underwent a PORP or TORP ossicular chain reconstruction, the improvement in AC and ABG is more outspoken (Table 6). In total, 69 cases had both complete tone audiometry and WRS and thus were included for the scattergram. The scattergram shows that most cases had minor improvement in PTA and some cases showed improvement in WRS (Figure 2A,B) The median dB necessary for 50% WRS showed minor improvement preoperatively to postoperatively, from 80 dB (IQR 70‐93) to 75 dB (IQR 62‐90, *P* = 0.026), respectively. Closure of the postoperative ABG ≤ 20 dB was possible in 9/42 cases with PORP/TORP reconstruction. Univariate analysis of 51 patients that underwent some form of ossicular chain reconstruction revealed no factors significantly associated with an ABG ≤ 20 dB postoperatively (Table 7).

**Table 5:** Audiometric Evaluation for All Patients With Complete Tone Audiometry (n = 78) (Median, IQR)

	Preoperative	Postoperative	*P* value
Bone conduction	23.8 (12.5‐36.3)	21.9 (13.6‐37.8)	0.348
Air conduction	56.9 (46.8‐73.8)	53.8 (38.8‐70.3)	<0.001^a^
ABG	32.3 (25.0‐45.0)	30.0 (22.0‐37.8)	0.004^a^

Values are in dB.

Abbreviations: ABG, air‐bone gap; IQR, interquartile range.

^a^ Significant using Wilcoxon signed‐rank test.

**Table 6:** Audiometric Evaluation for All Patients With Complete Tone Audiometry That Underwent a PORP or TORP Ossicular Chain Reconstruction (n = 42) (Median, IQR)

	Preoperative	Postoperative	*P* value
Bone conduction	23.1 (12.5‐37.8)	20.0 (13.8‐36.6)	0.210
Air conduction	63.1 (49.1‐73.8)	51.9 (35.9‐68.1)	<0.001^a^
ABG	34.4 (26.9‐46.3)	26.3 (21.3‐35.6)	0.002^a^

Values are in dB.

Abbreviations: ABG, Air‐bone gap; IQR, interquartile range; PORP, partial ossicular replacement prosthesis; TORP, total ossicular replacement prosthesis.

^a^ Significant using Wilcoxon signed‐rank test.

**Table 7:** Univariate Analysis of Possible Prognostic Factors for Postoperative ABG ≤ 20 dB in 51 Patients That Underwent Ossicular Chain Reconstruction (n)

	ABG ≤ 20 dB		
	Yes (n = 10)	No (n = 41)	*P* value
Age in years (median, IQR)	57 (37‐62)	51 (37‐61)	0.626
AC preoperatively in dB (median, IQR)	55.6 (36.9‐70.6)	61.3 (48.5‐72.5)	0.420
BC preoperatively in dB (median, IQR)	20.6 (10.0‐30.3)	21.3 (13.1‐39.4)	0.399
ABG preoperatively in dB (median, IQR)	37.5 (23.8‐47.2)	32.5 (27.5‐45.6)	0.943
Time since original CWD surgery in years (median IQR)	23.5 (16.8‐37.3)	24.5 (17.3‐37.3)	0.922
Any postoperative complication	1	5	1.000
Merchant grade preoperative 2‐3	9	37	1.000
Intact stapes	5	17	0.728
PORP/TORP reconstruction vs other types of ossicular chain reconstruction	9	33	0.667

Abbreviations: ABG, air‐bone gap; AC, air conduction; BC, bone conduction; CWD, Canal Wall Down; IQR, interquartile range; PORP, partial ossicular chain prosthesis; TORP, total ossicular chain prosthesis.

## Discussion

In this retrospective cohort study, we have presented the effectiveness and safety of secondary obliteration of troublesome radical cavities using S53P4 BAG. At the most recent follow‐up visit, 95% of the patients had a Merchant grade of 0 to 1, which is the main objective of secondary obliteration. Additionally, postoperative complications were only seen in 12 cases and they were seldom major. The main strengths of this study are the follow‐up time, the homogeneity in obliteration material and technique, and the fact that it is the first systematic article on S53P4 BAG for secondary obliteration of troublesome radical cavities.

Our study has demonstrated excellent dry ear rates as indicated by the Merchant grade at the most recent follow‐up visit. There are several articles to which our results should be compared to. First, Yung et al, in 2011, presented a case series on the use of hydroxyapatite, presenting a dry ear rate of 98% after 3 years.^7^ In this study, however, revision surgeries without obliteration and other types of surgery were included and all patients were operated by the same senior surgeon. In contrast, our study only included one type of procedure and patients were operated by 4 surgeons with varying levels of experience, although surgical participation rate was not equal among those four. Second, Patil et al, utilizing in part the same patients as Yung et al, presented a dry ear rate of 93.7% after 1 year.^3^ Third, Vercruysse et al, using the bony obliteration technique, presented a dry ear rate of 94%.^8^ Similar to Yung et al, all patients were operated by the same senior surgeon. Fourth, Van der Toom et al, in addition a dry ear rate of 96.4%, also showed the superiority of the obliteration technique over a nonobliterative technique in a retrospective comparative study.^1^ Unfortunately, all four studies solely presented a dry ear rate without the use of a standardized grading system. Therefore the comparability is somewhat diminished. Geerse et al using a standardized grading system, presenting a Merchant grade of 0 to 1 in 93%.^28^ Another limitation of the available literature is the inclusion of patients with cholesteatoma, confounding their results. Having a discharging ear due to cholesteatoma recidivism is different pathology than a discharging ear due to a troublesome radical cavity. Moreover, no grading system exists to determine severity of disease in troublesome radical cavities, making any comparison difficult.

Overall, our dry ear rate seems to be comparable to the available literature. This is especially important as the average time between original CWD and secondary obliteration was 25.3 years, showing the chronic nature of the symptoms of our patients. Although outpatient visits should not be fully ceased postoperatively in order to detect potential delayed problems, obliteration allows for the frequency of visits to be reduced to once per year or even less.

When investigating possible factors that would predict failure of the surgery, solely postoperative complications were found to be significantly associated with frequent postoperative otorrhea. Perhaps it shows that difficulties in the healing process result in surgical failure. No other variables such as time between original CWD surgery and secondary obliteration were significantly associated with surgical failure. The origin of postoperative otorrhea is possibly multifactorial and not all factors could be scored in this retrospective study, such as the frequency of previous surgeries performed. The rate of Merchant grade 2 was more than threefold higher eight weeks following surgery compared to the most recent follow‐up visit. This could be because the external auditory canal and tympanic membrane would still be healing 8 weeks postoperatively.

Few complications were seen and all except one were minor. This is especially relevant because surgery on troublesome radical cavities can be challenging. Most cavities had been purulent for years and exposed structures such as the facial nerves or labyrinth were seen in 37% of patients. Fortunately, no deaf ears or damage to the facial nerve was observed. The rate of revision surgeries is comparable to the currently available literature.^1^, ^3^, ^7^, ^28^ Only one patient developed a cholesteatoma postoperatively. No cholesteatoma was discovered under the obliteration material. It should be noted that in our hospital, a nonecho planar diffusion‐weighted magnetic resonance imaging is not part of the standard follow‐up of patients that underwent secondary obliteration. Therefore, it is possible that our one cholesteatoma is an underestimation.

Hearing rehabilitation was challenging. The overall improvement in AC and ABG was small and probably with limited clinical relevance. Similar results are found in other studies.^8^, ^28^ Fortunately, due to the high rates of dry ears after secondary obliteration, patients would again be able to wear conventional hearing aids as compensation for their hearing loss. The minor improvement can be explained in part by the fact that ossicular chain reconstruction was not possible in all patients. When solely evaluating patients with a PORP or TORP reconstruction, a clinically relevant improvement in AC and ABG was detected. There is some risk of selection bias, as reconstruction was only performed if the operator thought it was possible and beneficial. Another possible explanation is a higher incidence of stapes fixation due to the frequent years of otorrhea and purulence.^29^ Unfortunately, this information could not be retrieved from the patient files. Additionally, no factors were significantly associated with a postoperative ABG ≤ 20 dB, showing that other, unavailable, factors might predict the hearing outcome. It is important to counsel patients that the goal of the surgery is a dry and safe ear, and any hearing improvement is supplementary.

There are several limitations to be mentioned. First, the retrospective nature of the study limits the data validity. Nevertheless, as we choose to include all patients that underwent secondary obliteration in our institute, we think our study gives a realistic picture of the effectiveness of secondary obliteration using S53P4 BAG. As almost all patients since 2011 with troublesome radical cavities were treated similarly, there is minimal risk of selection bias. Second, there was no nonobliterative cohort to compare the obliteration technique to. As our hospital seldom performs revision surgery of radical cavities without obliteration, no adequate cohort could be formed. A prospective, randomized trial of obliterative versus nonobliterative techniques is necessary for a definitive conclusion. Third, no data was available on QoL. QoL should also be included in future studies to further identify the benefit of secondary obliteration beyond Merchant grading.

In conclusion, secondary obliteration of troublesome radical cavities using S53P4 BAG is safe and effective. Complications were seldom seen, even in these challenging cases. Even though patients suffered from otorrhea for an average of 25 years since the initial CWD surgery, secondary obliteration was successful in resolving the symptoms of 95% of them.

## Author Contributions

**Victor Kroon**, gathered the data, performed the analysis and wrote the first draft of the manuscript; **Steven W. Mes**, designed the study, performed analysis, and gave critical feedback on the manuscript. **Pepijn A. Borggreven**, designed the study, gave critical feedback on the manuscript; **Rick van de Langenberg**, designed the study, gave critical feedback on the manuscript; **David R. Colnot**, designed the study, gave critical feedback on the manuscript; **Jasper J. Quak**, designed the study, interpreted the data and gave critical feedback on the manuscript.

## Disclosures

### Competing interests

None.

### Funding source

Diakonessenhuis Research grant 2021, De Cornelis Visser stichting.
